# Supplementary material for: Visual motion perception as online hierarchical inference
Source: Nat Commun. 2022 Dec 1;13:7403. doi: 10.1038/s41467-022-34805-5 (PMC9715570; doi:10.1038/s41467-022-34805-5)
Supplement: Supplementary file 3 — Description of Additional Supplementary Files [file 41467_2022_34805_MOESM3_ESM.pdf]

Description of Additional Supplementary Files

File name: Supplementary Movie 1

Description: Johansson experiment.

Top: 3-dot motion display. *Bottom left:* Evolution of motion strengths, $\lambda_m(t)$. *Bottom right:* Motion sources, $s_m(t)$. The stimulus is decomposed into horizontal shared motion (magenta) and vertical individual motion (green) for the central dot.

File name: Supplementary Movie 2

Description: Duncker wheel.

Top: Duncker wheel motion display. *Bottom left:* Evolution of motion strengths, $\lambda_m(t)$. *Bottom right:* Motion sources, $s_m(t)$. The stimulus is decomposed into rightward shared motion (magenta) and clockwise rotation (green) for the dot on the rim.

File name: Supplementary Movie 3

Description: Motion illusion from Lorenceau (1996).

Motion displays without and with motion noise are presented. Multiple noise levels are used because the noise threshold for inducing the perceptual switch is participant-dependent. To prevent priming on a certain grouping, different random groups were drawn for every noise level, in this demo video. (The simulation in Fig. 5 used, apart from σ_{obs}^2, identical inputs.)

File name: Supplementary Movie 4

Description: Nested structure-from-motion displays.

A series of videos covering all SfM conditions from Fig. 6. The normal rotation speed is 90°/s, the faster speed is 135°/s. The outer cylinder is 50% larger than the inner cylinder, by radius. In the videos, points have an average lifetime of 250ms to preclude object-indexed tracking of dots.

File name: Supplementary Movie 5

Description: Location-indexed stimulus with varying fraction of shared motion.

Every 10s, the fraction of shared motion across the apertures increases by 1/8, ranging from $q = 1/8$ (almost independent motion) to $q = 7/8$ (almost fully correlated motion). Steps in q are marked by brief flashes of gray squares.
